# Bochdalek Hernia in an Adult Causing Intraoperative Complication

**DOI:** 10.7759/cureus.18010

**Published:** 2021-09-16

**Authors:** Kalvin Zee, Ashna Haque, Conor Kelly

**Affiliations:** 1 General Surgery, Rocky Vista University College of Osteopathic Medicine, Ivins, USA

**Keywords:** paraesophaegal hernia, pleuroperitoneal membrane, nissen fundoplication, pneumothorax, bochdalek hernia

## Abstract

Bochdalek hernias are rarely diagnosed in adults and account for 0.17-6% of all diaphragmatic hernias. It is a congenital diaphragmatic hernia caused by a defect in the posterior attachment of the diaphragm due to a failure of the pleuroperitoneal membrane closure in utero. This may rarely cause chest pain, respiratory symptoms, or gastrointestinal symptoms. In this study, we present a case of a laparoscopic paraesophageal repair via Nissen fundoplication. The incidental finding and subsequent repair of a Bochdalek hernia during this case may have resulted in complications of the surgery including pneumothorax due to the defect in the pleuroperitoneal membrane.

## Introduction

Bochdalek hernias are very rare, accounting for 0.17-6% of all diaphragmatic hernias [1]. They are due to failure of the pleuroperitoneal membrane to close in utero, causing a defect in the posterior attachment of the membrane to the diaphragm [2]. As a result, clinical manifestations usually arise in children [3] but can rarely go undiagnosed until it presents symptomatically in adults or via an incidental computed tomography (CT) finding. 

Clinical symptoms of Bochdalek hernias in adults include chest pain, respiratory symptoms, abdominal pain, and even features of intestinal obstruction including gastric volvulus [4,5]. Diagnosis of Bochdalek hernias includes CT imaging showing fat above the diaphragm with possible organ entrapment [6]. 

We report a case of an incidental Bochdalek hernia found during repair of a paraesophageal hernia with Nissen fundoplication. The subsequent repair of the Bochdalek hernia may have contributed to the development of pneumothorax and successive decompensation during the surgery. 

## Case presentation

**Figure 1:**
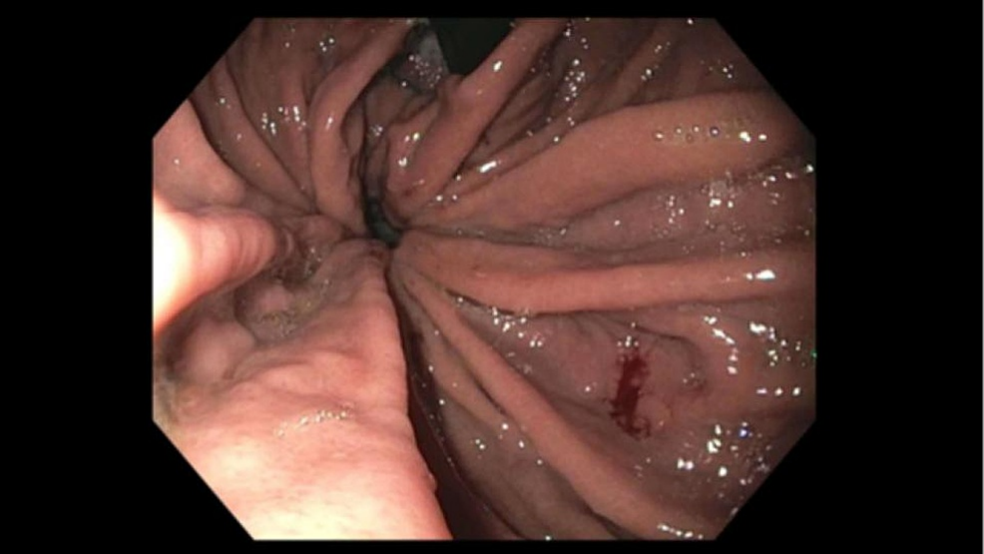
Esophagogastroduodenoscopy showing possible paraesophaegal hiatal hernia

The patient is a 62-year-old male (body mass index: 26.85) who presented to a surgical clinic with complaints of acid reflux for the past several years. The patient also had secondary complaints of cough, epigastric pain, and regurgitation during this time. The symptoms occurred nightly and worsened with laying down. He has tried proton pump inhibitors that helped slightly, as well as dietary modifications. An esophagogastroduodenoscopy (EGD) was performed in 2016, which showed distal esophageal ulceration and a 4-cm hiatal hernia. Biopsies taken at the time were significant for chronic esophagitis. Repeat EGD done one month before presentation to the surgical clinic showed the gastroesophageal junction at 35 cm with a 2-cm hiatal hernia and possible large paraesophageal hernia (Figure 1). Biopsy showed moderate gastritis. 

The patient has a past medical history of asthma, cavernous hemangioma of the liver, sleep apnea, gout, hypertension, and benign prostatic hypertrophy. He has a surgical history of esophageal dilation, cholecystectomy, and tonsillectomy. Home medications include allopurinol, amlodipine, dicyclomine, spironolactone, budesonide, albuterol, tamsulosin, and losartan. The patient has no known drug allergies. He has never used alcohol, tobacco, or any illicit substances. The patient was previously employed as a firefighter but has since retired. Family history is noncontributory. 

On physical examination, vitals were within normal limits with a blood pressure of 109/60, heart rate of 96 beats per minute, temperature of 36.1 Celsius, and physical examination was normal. An esophageal manometry study was performed, showing adequate peristalsis and 30% failed/dropped swallows. The patient was diagnosed with gastroesophageal reflux disorder (GERD) with hiatal hernia. A laparoscopic Nissen fundoplication with hiatal hernia repair with crural mesh reinforcement was discussed with the patient and he consented to the surgery. Risks and side effects including risk of esophageal and vagal nerve injury, bleeding, and infection were discussed, as well as anesthetic complications such as allergic reactions, pneumonia, deep vein thrombosis (DVT), myocardial infarction, and death. The patient voiced understanding and gave consent for the surgery. 

Surgery 

On the day of surgery, the patient was taken to the operating room and placed in a supine position. General anesthesia was induced, and the patient’s arms were placed on padded arm boards. PlexiPulse boots were used for DVT prophylaxis, and the abdomen was prepped from the nipple line to the pubis using Chlora Prep and draped in a sterile fashion. 

An incision was made in the left upper quadrant of the epigastrium overlying the lateral aspect of the rectus abdominus using a 5-mm bladeless trocar under direct trans-trocar visualization. A CO_2_ pneumoperitoneum was created to a pressure of 15 cm of H_2_O. The laparoscope was introduced along with three more 5-mm trocars placed under direct intraabdominal visualization in standard upper abdominal locations. A flexible liver retractor was placed through a small fascial defect to the left of the xiphoid process and held in place by a mechanical arm. The gastrohepatic ligament was then taken down using a harmonic scalpel. The exposed right crus was identified and the overlying peritoneum incised, entering the periesophageal space. The peritoneum was dissected away from the hiatus and the dissection was then carried across the midline anteriorly to the left. 

**Figure 2:**
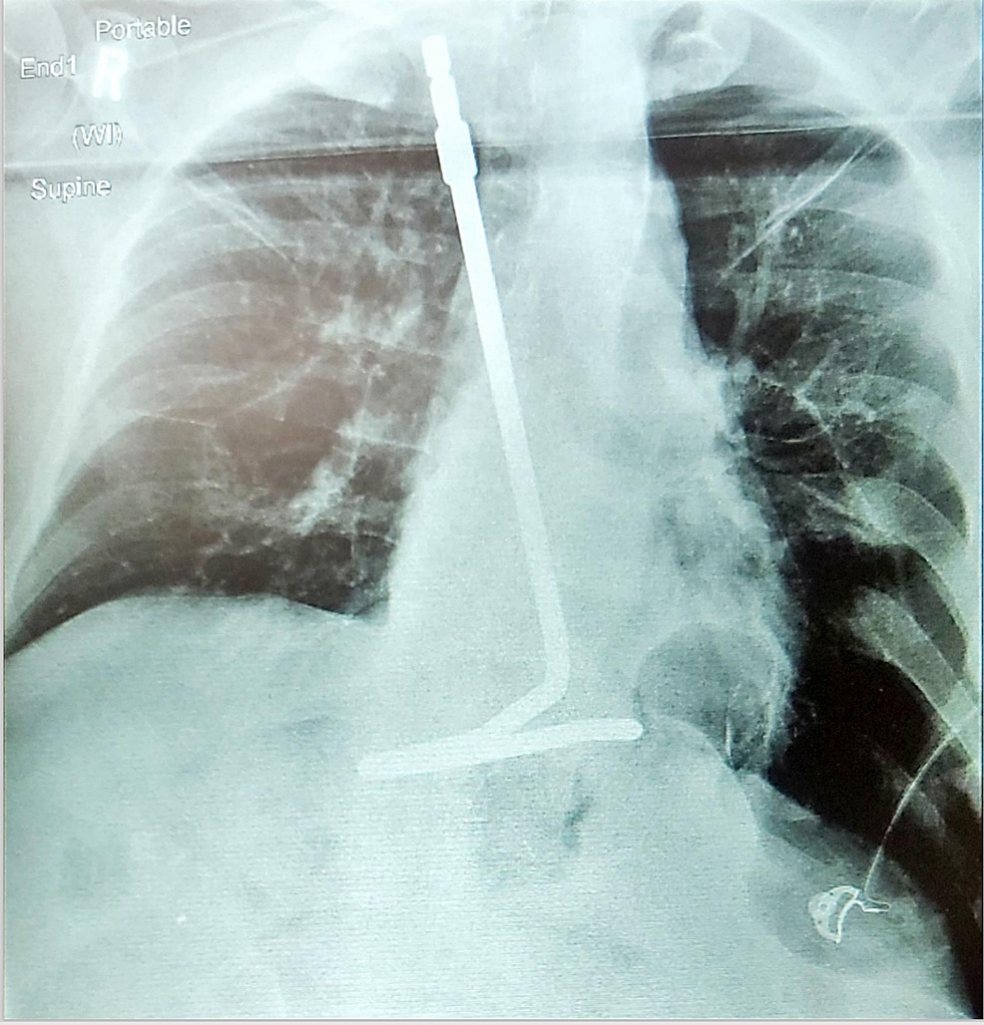
Left-sided pneumothorax on chest X-ray

At this point, a secondary defect in the diaphragm was found, which was determined to be a 4 cm x 2 cm Bochdalek hernia with an incarcerated cardia and 12 cm hernia sac. The cardia was dissected away from the diaphragm out of the second hernia defect. The Bochdalek hernia defect was then sutured shut with four #0 Ethibond sutures. During this process, the patient acutely decompensated and became hypotensive and hypoxic. The CO_2_ was released and the Trendelenberg position was reversed. A chest radiograph was obtained, which showed a moderate-sized pneumothorax (Figure 2). 

After the patient stabilized, insufflation at 10 cm H_2_O was reinstituted and the patient tolerated this with restoration of his oxygenation and blood pressure. The Bochdalek hernia repair was then completed before starting the fundoplication. The hiatus was closed using a series of four #0 Ethibond sutures. The crural repair was then reinforced with a 6 x 8 cm piece of Veritas bovine pericardial graft notched anteriorly to accommodate the esophagus. This was then secured using a series of secure strap anchors. Finally, the cardia was brought around behind the esophagus and a 360-degree fundoplication 1.5 cm long was created, securing the wrap with 3 #0 Ethibond sutures. The hiatal region was irrigated out and aspirated dry. Hemostasis was achieved and the wrap was inspected before removal of the Penrose drain. The trocars were then withdrawn, and CO_2_ was released. The skin wounds were then closed using subcuticular 4-0 Monocryl sutures with Dermabond. 

The patient was discharged to an intensive care unit for continued medical management of the pneumothorax and subsequently discharged from the hospital after two days with instructions to slowly advance diet consistency over the next month and avoid exertion. He was last seen in a follow-up in clinic two weeks later, where he reported resolution of his GERD with appropriately healing abdominal incisions. 

## Discussion

Bochdalek hernia is a type of congenital diaphragmatic hernia that occurs due to the failure of closure of the pleuroperitoneal folds causing a defect in posterolateral diaphragmatic formation [7]. This defect can lead to protrusion of abdominal contents into the thoracic cavity thereby interfering with lung development. Due to protrusion, the pathophysiology of the condition is severe cardiopulmonary complications including lung hypoplasia and cardiac dysfunction. This presents as severe cardiopulmonary complications in perinatal life like lung hypoplasia or pulmonary hypertension. The incidence of this condition is 0.8-5/10,000 births [7]. Rarely, the hernia can remain asymptomatic until adulthood where initial presentation can be pulmonary or abdominal complaints. In this case, the patient was completely asymptomatic until the Nissen fundoplication procedure when the hernia was found. 

Iatrogenic pneumothorax during laparoscopic surgery is a rare complication. However, it has been reported in various cases [8-11]. In this case, we believe that the reverse Trendelenburg position, preferred for the Nissen fundoplication, was a major contributing factor in allowing the CO_2_ used for insufflation to enter the thorax through the diaphragmatic Bochdalek defect. The rapid development of the resulting pneumothorax led to the hemodynamic collapse of the patient. The pneumothorax found ipsilaterally to the defect and the rapid improvement of hemodynamic factors upon lowering the head of the table, reducing insufflation pressures, and correcting the diaphragmatic defect all support an iatrogenic cause. 

Management of intraoperative pneumothorax includes discontinuing nitrous oxide and lowering the intraperitoneal pressure with the use of positive airway pressure [12]. This must be done immediately to prevent decreased surgical vision or cardiorespiratory instability [13]. Manual ventilation with 100% oxygen should be started immediately following decompensation. Imaging should then be obtained to assess the need for a chest tube. In the case of our patient, a chest tube was not needed as they restabilized with restoration of his oxygenation and blood pressure. However, in cases of continued decompensation, further intervention should be performed including chest tube and cessation of the surgery. 

## Conclusions

Intraoperative pneumothorax resulting from an unplanned Bochdalek hernia takedown and repair during a Nissen fundoplication can create a potentially deadly scenario. In the rare case of these factors coming together, we suggest that lower insufflation pressures be used, the head of the table to remain as close to level as possible, the takedown and repair of the diaphragmatic hernia to be completed as quickly as possible and ensure that the anesthesia team is notified to remain alert to any small changes or trends in hemodynamic parameters, especially surrounding the time of the hernia takedown. In the case of the development of an intraoperative pneumothorax with changes in hemodynamic parameters, we suggest that insufflation be turned off and gas allowed to escape through abdominal ports, rapid chest x-ray or ultrasound be obtained, and assessment for need of a chest tube be determined. 
